# PHL12/478: Cancer Information on the Internet: Same patients, new questions?

**DOI:** 10.2196/jmir.1.suppl1.e92

**Published:** 1999-09-19

**Authors:** B Hiller

**Affiliations:** ^1^German Cancer Information ServiceGerman Cancer Research CenterHeidelbergGermany

**Keywords:** Neoplasms, Patient Education, Patient Psychology, Internet, Electronic Mail

## Abstract

**Introduction:**

Since 1986, the German Cancer Information Service at the German Cancer Research Center has been working as a national telephone service, answering more than 155.000 inquiries from cancer patients and their families. In 1996, the service received the first E-mail requests, and in early 1999, a Web site with more than 1,8 MB information on cancer related topics was installed. More and more callers name the internet as one of their information resources. Who are these callers? Which questions do they ask? Are there specific problems caused by the internet information?

**Methods:**

In 1998, the questions of cancer patients and their families using electronic mail to contact the Cancer Information Service were compared to those of the callers. From April to September, 1999, a short interview was performed with callers identified as internet users, asking for the pros and cons and their special problems with their internet searches.

**Results:**

Cancer patients with access to the internet tend to use as many sources of information as possible and rate them differently. The internet is most valued for very specific information otherwise not available to patients, but only to health professionals. Treatment options are the main topic of interest. Patients and their families, however, still ask the Cancer Information Service for assistance in assessing the relevance of the news for their own situation, especially when clinical trials or unconventional methods are concerned. E-mail questions to the Cancer Information Service do not differ from those asked in phone calls.

**Discussion:**

Internet is developing into a new and powerful instrument to give patients access to cancer information. However, problems are caused by the number and diversity of Web sites presenting medical contents, the anonymity of many pages and the commercial aspects of information. Cancer organizations, i.e. hospitals, research centers and governmental institutions, should take the patients' needs into account in designing their Web sites. Guidelines, standards and methods of quality control could be helpful and should be established.

